# A novel evidence combination method based on stochastic approach for link-structure analysis algorithm and Lance-Williams distance

**DOI:** 10.7717/peerj-cs.1307

**Published:** 2023-04-18

**Authors:** Qi Tang, Jianyu Xiao, Kefeng Wu

**Affiliations:** School of Computer Science and Technology, Huaibei Normal University, HuaiBei, AnHui, China

**Keywords:** D-S combination rule, SALSA algorithm, Lance-Williams distance, Correlation coefficient

## Abstract

In response to the traditional Dempster–Shafer (D-S) combination rule that cannot handle highly conflicting evidence, an evidence combination method based on the stochastic approach for link-structure analysis (SALSA) algorithm combined with Lance-Williams distance is proposed. Firstly, the degree of conflict between evidences is calculated based on the number of correlation coefficients between evidences. Then, the evidences with a number of correlation coefficients greater than the average number of correlation coefficients of evidence are connected to construct an evidence association network. The authority weight of the evidence is calculated based on the number of citations in the concept of SALSA algorithm combined with the support of the evidence. Subsequently, the Lance-Williams distance between the evidences is calculated and transformed into support of the evidence. Next, the authority weight and support of evidence are combined to jointly construct a novel correction coefficient to correct the evidence. Finally, the corrected evidence is fused using the D-S combination rule to obtain the final fusion result. The numerical results verify that the method proposed in this paper can effectively solve the problem of the traditional D-S combination rule being unable to handle highly conflicting evidence.

## Introduction

The Dempster–Shafer (D-S) theory of evidence (Petturiti & Vantaggi, 2017) was first proposed by Harvard mathematician A.P. Dempster in the 1960s. A series of articles were published in 1967, marking the birth of evidence theory. It was further refined by his student Shafer, who introduced the concept of “trust function” and established a mathematical approach to uncertainty inference based on “evidence” and ”combination” (Shafer, 2020). The theory uses upper and lower probabilities to deal with uncertainty in the problem, and the D-S combination rule can fuse human predictions, sensor transmitted data, and classifier outputs. Currently, the D-S evidence theory has been widely used in the areas of fault diagnosis (Ding, Hou & Ding, 2019; Bo et al., 2021), risk assessment (Zhang et al., 2009), military commands (Xu et al., 2018), legal case analysis, medical diagnosis, and target identification.

In practical applications, the D-S combination rule is incomplete and unable to handle highly conflicting evidence to the extent that it produces results that are contrary to the facts. Several scholars in China and abroad have studied and improved this deficiency, and their methods are mainly divided into two categories. The first category is the revision of evidence sources. This approach considers that the traditional D-S combination rule is not problematic in itself, but requires processing of the original evidence. An example is the weighted average method proposed by Murphy (2000), who proposed a reasonably easy way to deal with conflict of evidence under the priority of average confidence. First, the arithmetic mean of the basic probability assignment of all evidence is found, and then, the traditional D-S combination rule is used for sub-fusion. The shortcoming of this method is that it uses only a simple weighted average of the evidence without further consideration of the interrelationship between evidences. Deng et al. (2005) introduced the Jousselme distance function to measure the relationship between evidences based on Murphy, and fused the evidence after weighted averaging; this method can identify the target when there is highly conflicting evidence. Hu et al. (2009) proposed to deal with conflicting evidence based on the weight coefficient of modified evidences and defined a new measure of evidence conflict through Pignistic transformation, which can effectively deal with highly conflicting evidence. The second case is the modification of the combination rule. This method considers that the fusion results produce contradictory facts due to the deficiency of the combination rule itself. The most representative example is the Yager method (Yager, 1987), in which Yager amended the combination rule by assigning completely conflicting information to an uncertain domain. However, the modified rule did not yield satisfactory results when fusing more than two evidence sources. Sun, Ye & Gu (2000) argues that highly conflicting evidence is also partly available information, a new combination rule based on Yager is proposed to make the result of fusion of conflicting evidence more desirable. However, the obvious shortcoming of this method is that the authors treat the credibility of the collective evidence as the credibility of the individual evidence. Li et al. (2002) assigned the probability of supporting conflicting evidence according to the average support of each proposition, and the fusion of highly conflicting evidence was able to obtain the desired result; yet, this method ignored the credibility of the evidence.

In this paper, based on the improved method of modifying the evidence source, we propose to combine the stochastic approach for link-structure analysis (SALSA) algorithm (Lempel & Moran, 2000) with the Lance-Williams distance to jointly construct a novel correction factor for the evidence.

The SALSA algorithm is a new link analysis algorithm proposed by Lempel & Moran (2000), which combines the main features of PageRank (Brin & Page, 1998) and Hyperlink-Induced Topic Search (HITS) (Kleinberg, 1999) algorithms. The SALSA algorithm contains the two parts of authority (Authority) value and hub (Hub) value of the HITS algorithm and the Markov chain in the PageRank algorithm, which is currently one of the best link analysis algorithms. According to the concept of the SALSA algorithm, the number of citations is closely related to the authority value in the algorithm. In the evidence theory, the number of citations can be regarded as the extent to which an evidence is supported by other evidence. This paper combines the idea of the SALSA algorithm to calculate the weight of evidence. In the improvement of the D-S evidence theory, many scholars used a single correction coefficient to correct the evidence in order to make the calculated weight value of evidence more accurate. In this study, a compound correction coefficient is adopted to correct the evidence using the SALSA algorithm to calculate the authority value and combine the support degree of evidence to jointly determine the correction coefficient of evidence.

This paper focuses on solving the problem that the D-S evidence theory cannot deal with highly conflicting evidence by correcting the evidence sources. Firstly, we construct an evidence association network based on the correlation coefficients between the evidences, use the SALSA algorithm and combine the support of the evidence to jointly determine the correction coefficients of the evidence. Finally, we use the D-S combination rule to fuse the corrected evidence to obtain the final fusion results.

## D-S Theory of Evidence

### Basic concepts of D-S evidence theory

#### Basic probability assignment

Θ is a complete set of mutually incompatible fundamental propositions. The entire set of its subsets, *i.e.,* the power set of Θ is 2^Θ^; m is a function of 2^Θ^ on to $ \left[ 0,1 \right] $ satisfying the following:

1. the basic probability of an improbable event is 0, *i.e.*, *m*(∅) = 0;
2. The basic probability sum of all subsets in 2^Θ^ is 1, *i.e.*, ∀*A* ∈ 2^Θ^, *m*(*A*) ≥ 0 and ∑_*A*∈Θ_*m*(*A*) = 1.

*m*(*A*) is called the basic probability assignment (BPA) on Θ, also known as mass function. If *m*(*A*) ≠ 0, A is called a focal element. In reasoning, Θ is usually called frame of discernment, which represents a finite set of basic propositions of all possible conclusions under condition E. A subset of Θ, *i.e.,* the elements in 2^Θ^, can be understood as a proposition.

#### Belief function

The belief function is the minimum value of the total trust that supports A. In the frame of discernment Θ, the trust function based on BPA *m* is defined as follows:

(1) \begin{eqnarray*}Bel(A)=\sum _{B\subseteq A}m(B).\end{eqnarray*}

#### Plausibility function

Plausibility function means that it does not deny A ’s trust in you, and it is the maximum of the total trust that supports A. At the frame of discernment, the likelihood function based on BPA *m* is defined as follows:

(2) \begin{eqnarray*}pl(A)=1-Ble(\bar {A})=\sum _{B\cap A=\varnothing }m(B).\end{eqnarray*}

#### Confidence interval

In evidence theory, for a certain hypothesis A of frame of discernment $\Theta , \left[ Bel(A),Pl(A) \right] $ represents the confidence interval for A, which is used to indicate the degree of confirmation of a certain hypothesis.

#### D-S combination rule

The D-S combination rule for a limited number of mass functions *m*_1_, *m*_2_, …, *m*_*n*_ on the ∀*A*⊆Θ frame of discernment Θ are as follows:

(3) \begin{eqnarray*}({m}_{1}\oplus {m}_{2}\oplus \cdots \oplus {m}_{n})(A)= \frac{1}{1-K} \sum _{{A}_{1}\cap {A}_{2}\cap \ldots \cap {A}_{n}=A}{m}_{1}({A}_{1})\times {m}_{2}({A}_{2})\times \ldots \times {m}_{n}({A}_{n})\end{eqnarray*}

and

(4) \begin{eqnarray*}K=\sum _{{A}_{1}\cap {A}_{2}\cap \ldots \cap {A}_{n}=\varnothing }{m}_{1}({A}_{1})\times {m}_{2}({A}_{2})\times \ldots \times {m}_{n}({A}_{n})\end{eqnarray*}

where *K* is called the conflict factor, $K\subseteq \left[ 0,1 \right] $. When *K* → 1, the conflict between evidences is large; when *K* → 0, the conflict between evidences is small. If *K* = 1, there is complete conflict between evidences, and *K* = 0 indicates that there is no conflict between evidences.

### Challenges of D-S evidence theory

Due to the diversity and complexity of data, evidence fusion using the D-S evidence theory may lead to situations contrary to the facts, which are mainly divided into the following four categories.

#### Full conflict paradox

The basic probability distribution functions of frame of discernment Θ = *A*, *B* and evidence *E*_1_, *E*_2_ are:

\begin{eqnarray*}\begin{array}{@{}l@{}} \displaystyle {m}_{1}(A)=1,{m}_{1}(B)=0;\\ \displaystyle {m}_{2}(A)=0,{m}_{2}(B)=1. \end{array} \end{eqnarray*}

In this example, according to common sense, the combination result is 1 > *m*(*A*) = *m*(*B*) > 0. The conflict factor *K* = 1, and *E*_1_, *E*_2_ are calculated by Eq. (4) and the two evidences are in complete conflict, which cannot be synthesized using the traditional D-S evidence theory. This type of conflict is called the full conflict paradox.

#### 0 trust paradox

The basic probability distribution functions of the frame of discernment Θ = {*A*, *B*, *C*} and evidence *E*_1_, *E*_2_, *E*_3_ are:

\begin{eqnarray*}\begin{array}{@{}l@{}} \displaystyle {m}_{1}(A)=0.9,{m}_{1}(B)=0.1,{m}_{1}(C)=0;\\ \displaystyle {m}_{2}(A)=0,{m}_{2}(B)=0.1,{m}_{2}(C)=0.9;\\ \displaystyle {m}_{3}(A)=0.7,{m}_{3}(B)=0.1,{m}_{3}(C)=0.2;\\ \displaystyle {m}_{4}(A)=0.8,{m}_{4}(B)=0.1,{m}_{4}(C)=0.1; \end{array} \end{eqnarray*}

In this example, the basic probability assignment of focal element A is generally high, followed by focal element C; finally, the basic probability assignment of focal element B is generally low. According to common sense, the combination result is 1 > *m*(*A*) > *m*(*C*) > *m*(*B*) > 0,  and the final result obtained by using the traditional D-S evidence theory is *m*(*A*) = *m*(*C*) = 0, *m*(*B*) = 1. The recognition target does not point to focal elements A and C with generally high basic probability assignment but points to focal element B with generally low basic probability assignment. The result is contrary to the fact, and this type of conflict is called the 0 trust paradox.

#### 1 trust paradox

The basic probability distribution functions of frame of discernment Θ = {*A*, *B*, *C*, *D*} andevidence *E*_1_, *E*_2_, *E*_3_are:

\begin{eqnarray*}\begin{array}{@{}l@{}} \displaystyle {m}_{1}(A)=0.99,{m}_{1}(B)=0.01;\\ \displaystyle {m}_{2}(B)=0.01,{m}_{2}(C)=0.99;\\ \displaystyle {m}_{3}(B)=0.01,{m}_{3}(D)=0.99. \end{array} \end{eqnarray*}

In this example, the basic probability assignment of evidence *E*_1_, *E*_2_, *E*_3_ to focal element B is low, and there is a high degree of trust in focal elements A, C and D, respectively, *i.e.,* all of them are 0.99. According to common sense, the combination result is 1 > *m*(*A*) = *m*(*C*) = *m*(*D*) > *m*(*B*) > 0,  and using the traditional D-S evidence theory, the final result is *m*(*A*) = *m*(*C*) = *m*(*D*) = 0, *m*(*B*) = 1. This type of conflict is called 1 trust paradox.

#### Evidence failure paradox

The basic probability distribution functions of frame of discernment Θ = {*A*, *B*, *C*, *D*} and evidence *E*_1_, *E*_2_, *E*_3_ are:

\begin{eqnarray*}\begin{array}{@{}l@{}} \displaystyle {m}_{1}(C)=0.35,{m}_{1}(C\cup D)=0.65;\\ \displaystyle {m}_{2}(B)=0.8,{m}_{2}(\Theta )=0.2;\\ \displaystyle {m}_{3}(B)=0.8,{m}_{3}(\Theta )=0.2. \end{array} \end{eqnarray*}

In this example, the evidence *E*_2_, *E*_3_ basic probability distribution function is the same; *E*_1_, *E*_2_ and *E*_3_ of the focal element are not the same and *m*_2_(*B*) = *m*_3_(*B*) > *m*_1_(*C*∪*D*) > *m*_1_(*C*). Therefore, according to common sense, the combination result should be 1 > *m*(*B*) > *m*(*C*∪*D*) > *m*(*C*) > 0, where *m*(*B*) < *m*_2_(*B*) = *m*_1_(*B*), *m*(*C*∪*D*) < *m*_1_(*C*∪*D*), *m*(*C*) < *m*_1_(*C*). The probability of pointing to the focal element B should be greater after adding evidence *E*_3_ again; then, *m*(*B*) increases, and the result obtained by synthesizing evidence *E*_1_, *E*_2_ using the traditional D-S evidence theory *m*(*C*) = 0.35, *m*(*C*∪*D*) = 0.65 is the same as *E*_1_. The final result obtained by synthesizing evidence *E*_1_, *E*_2_, *E*_3_ is still *m*(*C*) = 0.35, *m*(*C*∪*D*) = 0.65 (same as *E*_1_), evidence *E*_3_ fails, and the result is contrary to common sense. This type of conflict is called the evidence failure paradox.

## The Proposed Evidence Combination Method Based on Salsa Algorithm and Lance-Williams Distance

**Figure 1:**
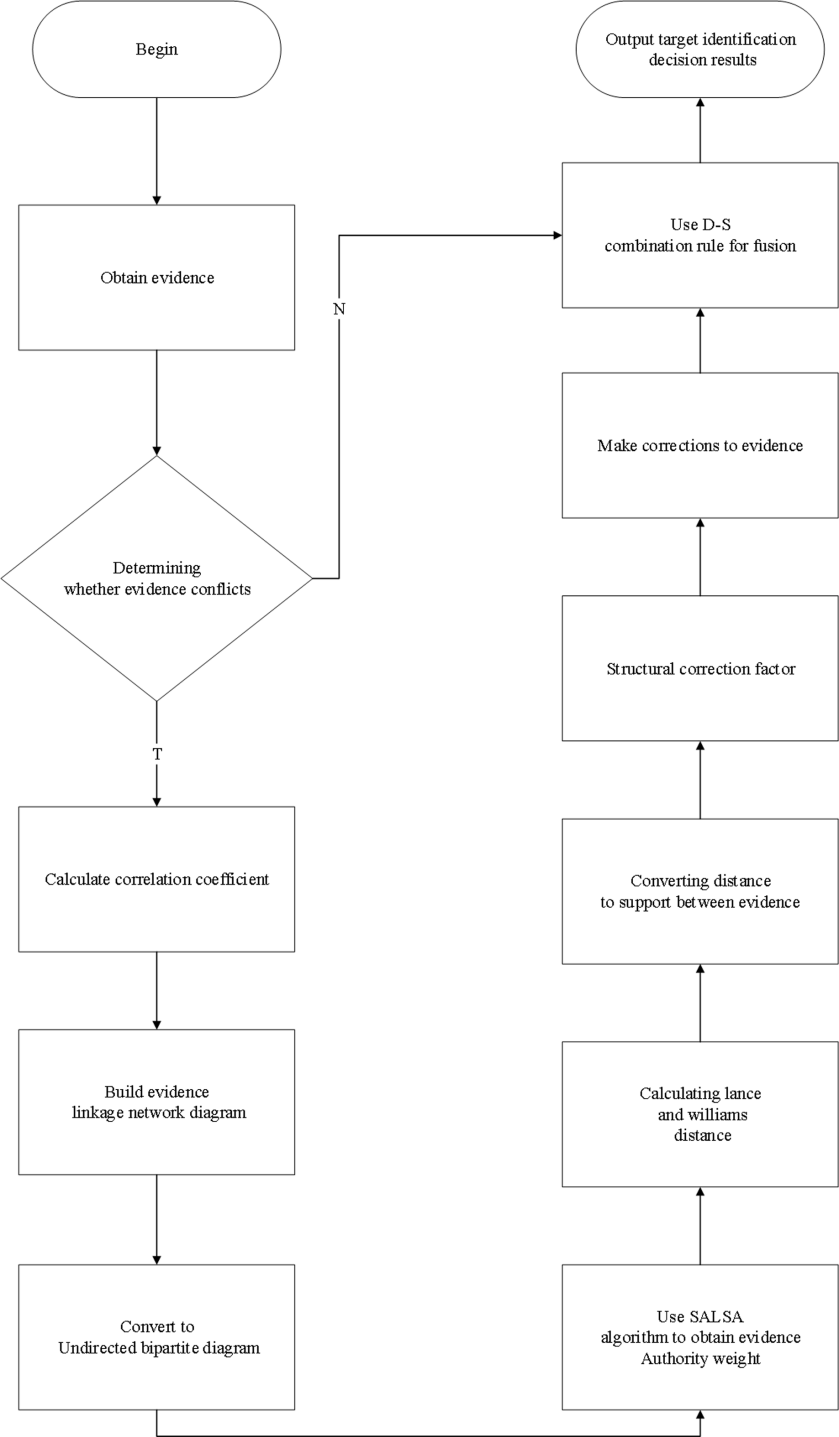
Specific steps flow chart.

Considering that the traditional D-S evidence theory cannot deal with highly conflicting evidence and produces contradiction with facts in practical applications. This paper uses the SALSA algorithm combined with Lance-Williams distance to jointly construct a novel correction coefficient to correct the evidence and use the D-S combination rule to fuse the corrected evidence in the following process. The flow chart of specific steps is shown in Fig. 1.

### Construction of network of evidence linkages

The evidence association network is a network that can indicate the correlation between evidences. The lower the conflict between two pieces of evidence, the stronger the correlation between evidences and the higher the similarity between the two pieces of evidence. Conversely, the higher the conflict between two pieces of evidence, the weaker the relevance of the evidence and the lower the similarity between evidences. The current methods to measure the degree of conflict between evidences include the amount of evidence agreement and conflict, conflict coefficient, evidence distance, information entropy, and correlation coefficient. Through analysis and comparison, the conflict coefficient and amount of consistency and conflict between evidence cannot accurately measure the degree of conflict between evidence in some cases. Additionally, the evidence distance mainly describes the degree of difference between evidences, which is not equivalent to describing the degree of conflict between evidences. In this paper, we use the correlation coefficient to measure whether there is a conflict between two pieces of evidence. Its specific definition can be found in the study presented in Yong, Li & Zhang (2011), and its calculation formula is described as follows.

The distributions of the random variables X and Y are as follows:

\begin{eqnarray*}X= \left( \begin{array}{@{}l@{}} \displaystyle {a}_{1}\ldots {a}_{k}\\ \displaystyle {p}_{1}\ldots {p}_{k} \end{array} \right) ,Y= \left( \begin{array}{@{}l@{}} \displaystyle {b}_{1}\ldots {b}_{k}\\ \displaystyle {q}_{1}\ldots {q}_{k} \end{array} \right) . \end{eqnarray*}

The bias entropy of the random variable X with respect to Y is defined as follows:

(5) \begin{eqnarray*}{H}_{Y}(X)=\sum _{k=1}^{n}{q}_{k}\times {e}^{-5{q}_{k}}\end{eqnarray*}

The entropy of association between the random variables X and Y is defined as follows:

(6) \begin{eqnarray*}H(X,Y)={H}_{Y}(X)+{H}_{X}(Y)\end{eqnarray*}

The partial correlation coefficients and correlation coefficients of the random variables are defined as follows:

(7) \begin{eqnarray*}{r}_{Y}(X)& = \frac{H(Y)}{{H}_{Y}(X)} \end{eqnarray*}

(8) \begin{eqnarray*}{r}_{X}(Y)& = \frac{H(X)}{{H}_{X}(Y)} \end{eqnarray*}

(9) \begin{eqnarray*}r(X,Y)& = \frac{H(X)+H(Y)}{{H}_{Y}(X)+{H}_{X}(Y)} .\end{eqnarray*}

The formula uses a similar idea to that of entropy, and the proposed concept of entropy-like is to vary the number of 0-1 over a wide range, because e and log are related by an inverse function, so the function e is used instead of log. But the simple *e*^−*p*_*k*_^ can not completely change the number of 0-1 in a wide range, which needs to add a coefficient on top of *e*^−*p*_*k*_^ into the form of *e*^−5*p*_*k*_^, this coefficient is closer to the log result based on the check calculation.

When the number of correlation coefficients between two pieces of evidence is large, it indicates that the evidence is highly correlated and less conflicting. If the correlation coefficient between two pieces of evidence is small, it indicates that the evidence is less correlated and more conflicting. The calculation of the full conflict paradox example using the correlation coefficient method yields *r* = 1; this indicates that the two pieces of evidence are consistent, there is no conflict, and the calculation is consistent with the facts.

The evidence involved in the combination is calculated by the above method to calculate the number of correlation links; the evidence with the number of correlation links greater than the average of the number of correlation links of that evidence is connected to build the evidence correlation network. The main idea of the evidence association network is that a high quality evidence is pointed towards by many high quality evidences, and points to many high quality evidences as well.

### Authority weights of evidence using SALSA algorithm

The SALSA algorithm is one of the link analysis algorithms which combines the main features of PageRank and HITS algorithms. According to the concept of SALSA algorithm, the evidence association network is transformed into an undirected bipartite graph, and the weights of the evidence are calculated using the formula of authority weights in SALSA algorithm. Then, the weights of the ith evidence *E*_*i*_ are calculated as follows:

(10) \begin{eqnarray*}{w}_{i}= \frac{ \left\vert {A}_{i} \right\vert }{ \left\vert A \right\vert } \frac{ \left\vert {B}_{ \left( i \right) } \right\vert }{ \left\vert {B}_{j} \right\vert } \end{eqnarray*}

where *i*, j =1 , 2, …, *N*,  *N* is the number of evidence, *A*_*i*_ is the number of evidence in the connected graph, *A* is the number of evidence in the authority subset, ${B}_{ \left( i \right) }$ is the number of incoming chains of evidence nodes, and *B*_*j*_ is the total number of incoming chains in the connected graph.

The number of evidences in the authority subset is $ \left\vert A \right\vert $. This factor is the same for any evidence node in the authority subset; thus, it does not affect the final ranking of the weights, serves to ensure that the weights are between 0 and 1, and can represent the weights in the form of probabilities. The higher the number of incoming chains $ \left\vert {B}_{ \left( i \right) } \right\vert $of evidence *E*_*i*_ in the connectivity graph, the higher number of evidence associated with that evidence, and the higher its importance. The greater the ratio $ \frac{ \left\vert {B}_{ \left( i \right) } \right\vert }{ \left\vert {B}_{j} \right\vert } $ between the number of incoming chains $ \left\vert {B}_{ \left( i \right) } \right\vert $ and the total number of incoming chains $ \left\vert {B}_{i} \right\vert $ contained in the connected graph of evidence *E*_*i*_, the greater the importance of this evidence in the evidence associated with it and the greater the corresponding weight.

### Use of Lance-Williams distance to obtain evidence support

Lance-Williams distance is a quantity that is both dimensionless and unitless. Thus, its numerical magnitude is independent of the chosen unit, overcoming the drawback that Ming’s distance is related to the magnitude of each indicator, while the dimensionless quantity is more suitable for expressing the distance between evidence. The Lance-Williams distance formula is introduced to calculate the distance between individual pieces of evidence, and the distance is transformed into the support of evidence. The specific steps of the method are described as follows.

After defining the identification frame Θ = {*A*_1_, *A*_2_, …, *A*_*n*_}, Lance-Williams distance between evidence *m*_*i*_ and *m*_*j*_ is as follows:

(11) \begin{eqnarray*}d({m}_{i},{m}_{j})= \frac{1}{N} \sum _{x=1}^{N} \frac{ \left\vert {m}_{ix}-{m}_{jx} \right\vert }{ \left( {m}_{ix}+{m}_{jx} \right) } .\end{eqnarray*}

The distance between individual pieces of evidence can be expressed as a distance matrix:

\begin{eqnarray*}D= \left( \begin{array}{@{}ccc@{}} \displaystyle 0&\displaystyle \cdots &\displaystyle {d}_{1n}\\ \displaystyle \vdots &\displaystyle \ddots &\displaystyle \vdots \\ \displaystyle {d}_{n1}&\displaystyle \cdots &\displaystyle {d}_{nn} \end{array} \right) \end{eqnarray*}

The similarity between individual pieces of evidence can be expressed in terms of the distance between the evidences:

(12) \begin{eqnarray*}{s}_{ij}=s({m}_{i},{m}_{j})=1-d({m}_{i},{m}_{j}).\end{eqnarray*}

When *s*_*ij*_ is larger, the similarity between the evidence is higher; the smaller the *s*_*ij*_, the lower the similarity between evidences. The degree of similarity of the evidence can be expressed by a similarity matrix as follows:

\begin{eqnarray*}S= \left( \begin{array}{@{}ccc@{}} \displaystyle 1&\displaystyle \cdots &\displaystyle {s}_{1n}\\ \displaystyle \vdots &\displaystyle \ddots &\displaystyle \vdots \\ \displaystyle {s}_{na}&\displaystyle \cdots &\displaystyle 1 \end{array} \right) . \end{eqnarray*}

The support of the evidence is calculated as *SUP*_*i*_; if two pieces of evidence are similar, the two pieces of evidence support each other. The higher the degree of similarity, the greater the degree of support. The degree of support of evidence is defined as follows:

(13) \begin{eqnarray*}SU{P}_{i}& = \frac{{R}_{i}}{\sum _{i=1}^{N}{R}_{i}} \end{eqnarray*}

(14) \begin{eqnarray*}{R}_{i}& =\sqrt{\sum _{j=1,i\not = j}^{N}(1-{d}_{ij})^{2}}.\end{eqnarray*}

### Correction and integration of evidence

To avoid that a single discount factor is not accurate enough to correct the evidence, this paper adopts a compound discount factor, and combines the SALSA algorithm concept with the support of evidence to jointly construct a new type of correction coefficient to correct the evidence. The correction factor *ω*_*i*_ for the ith evidence is defined as follows:

(15) \begin{eqnarray*}{\omega }_{i}={w}_{i}\times SU{P}_{i}.\end{eqnarray*}

Normalizing the correction factor is defined as follows:

(16) \begin{eqnarray*}{\omega }_{i}= \frac{{\omega }_{i}}{\sum _{i=1}^{N}{\omega }_{i}} .\end{eqnarray*}

The correction of the underlying probability assignment for each piece of evidence is defined as follows:

(17) \begin{eqnarray*} \left\{ \begin{array}{@{}l@{}} \displaystyle {m}_{i}^{{}^{{^{\prime}}}}({E}_{i})={\omega }_{i}\times {m}_{i}({E}_{i})\\ \displaystyle {m}_{i}^{{}^{{^{\prime}}}}(\Theta )=(1-{\omega }_{i})+{\omega }_{i}\times {m}_{i}(\Theta ) \end{array} \right. \end{eqnarray*}

The corrected evidence was fused using the D-S combination rule to obtain the final fusion results.

## Results and Analysis

To verify that the method proposed in this paper can deal better with highly conflicting evidence and solve the main problems existing in the traditional D-S evidence theory, this chapter cites single conflict and multi-conflict arithmetics to verify it. Additionally, the proposed method is used to synthesize several typical conflicting evidences to compare and analyze them with other combination methods. The purpose is to show that the proposed method can effectively solve the existing problems in the combination of highly conflicting evidence as well as the traditional D-S evidence theory.

### Comparative analysis of synthetic results of single conflict evidence

For the frame of discernment Θ = {*A*, *B*, *C*}, a sensor collects five mutually independent evidences whose corresponding basic probability assignments are as follows:

\begin{eqnarray*} \left\{ \begin{array}{@{}l@{}} \displaystyle {E}_{1}:{m}_{1}(A)=0.5,{m}_{1}(B)=0.2,{m}_{1}(C)=0.3\\ \displaystyle {E}_{2}:{m}_{2}(A)=0,{m}_{2}(B)=0.9,{m}_{2}(C)=0.1\\ \displaystyle {E}_{3}:{m}_{3}(A)=0.55,{m}_{3}(B)=0.1,{m}_{3}(C)=0.35\\ \displaystyle {E}_{4}:{m}_{4}(A)=0.55,{m}_{4}(B)=0.1,{m}_{4}(C)=0.35\\ \displaystyle {E}_{5}:{m}_{5}(A)=0.6,{m}_{5}(B)=0.1,{m}_{5}(C)=0.3 \end{array} \right. \end{eqnarray*}

Data source from literature (Tian, Ye & Wan, 2021).

**Figure 2:**
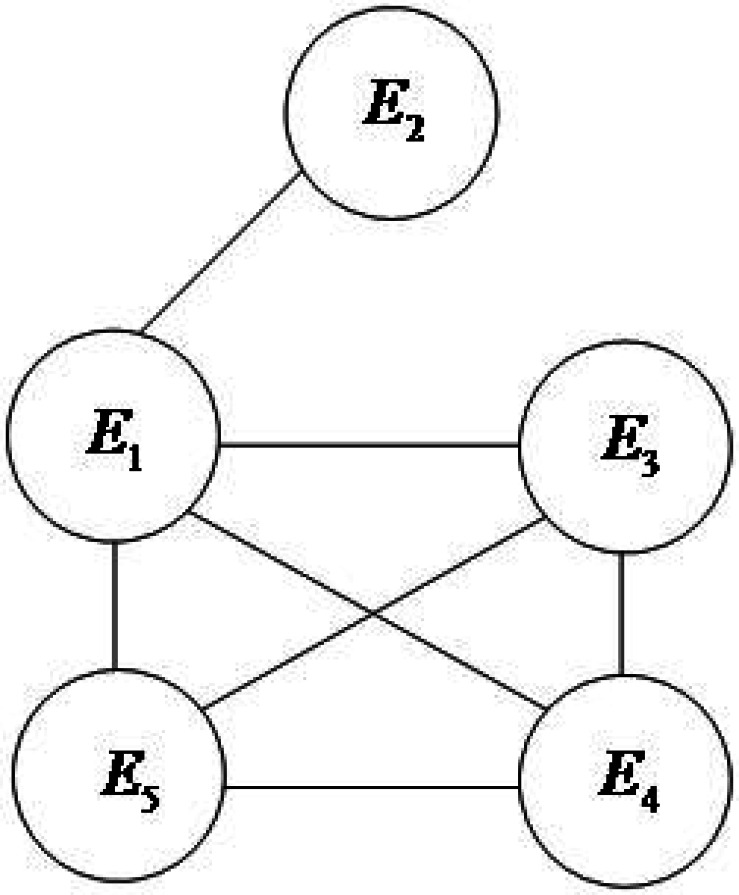
Evidence linkage network diagram.

The evidence association network is constructed according to the method described in section 3.1, as shown in Fig. 2.

**Figure 3:**
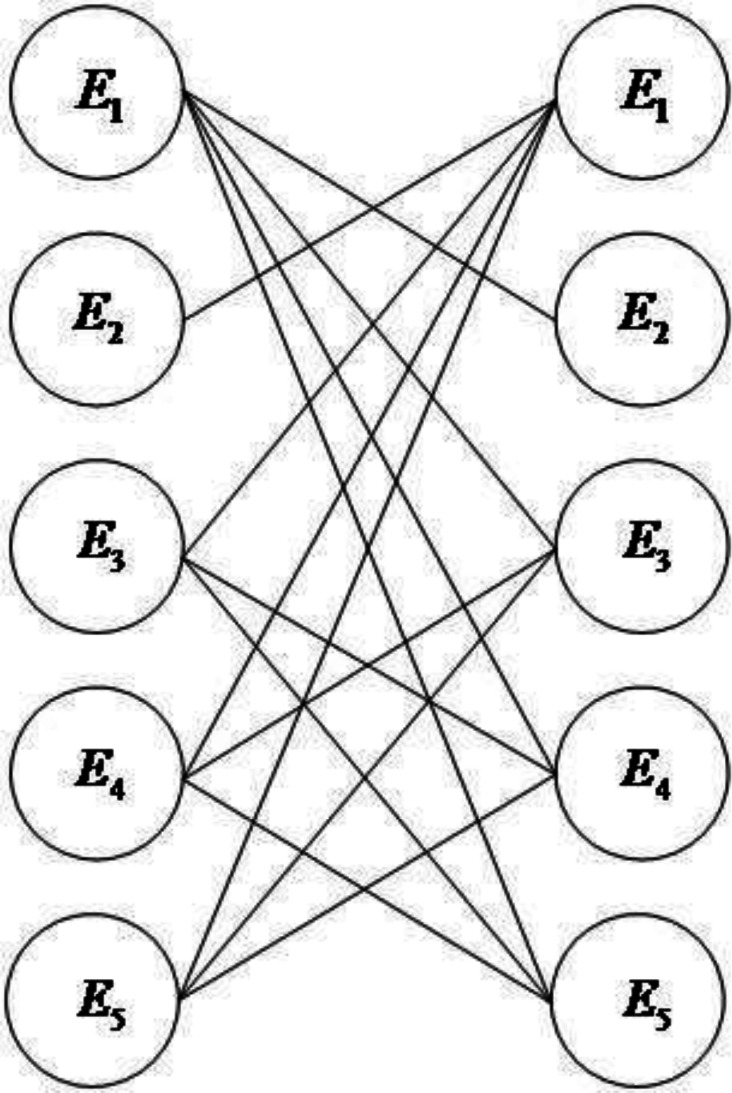
Undirected bipartite diagram.

We convert the evidence association network into an undirected bipartite graph, as shown in Fig. 3.

The authority weight of the evidence is calculated according to Eq. (10) as follows:

\begin{eqnarray*}w=[ \frac{16}{70} \frac{4}{50} \frac{15}{70} \frac{15}{70} \frac{15}{70} ]^{T}. \end{eqnarray*}

The support of the evidence can be calculated according to Eqs. (11)–(14) as follows:

\begin{eqnarray*}R=[0.2182\,0.0696\,0.2381\,0.2381\,0.2360]^{T}. \end{eqnarray*}

The correction factor for the evidence is calculated from Eqs. (15)–(16) as follows:

\begin{eqnarray*}\omega =[0.2398\,0.0265\,0.2453\,0.2453\,0.2430]^{T}. \end{eqnarray*}

From Eq. (17), the discounted basic probability assignment can be calculated as follows:

\begin{eqnarray*}\begin{array}{@{}l@{}} \displaystyle {\Theta }^{{^{\prime}}}=\{ \{ {A}^{{^{\prime}}}\} ,\{ {B}^{{^{\prime}}}\} ,\{ {C}^{{^{\prime}}}\} \} \\ \displaystyle {m}^{{^{\prime}}}=\{ 0.5355\,0.1452\,0.3193\} . \end{array} \end{eqnarray*}

Finally, the amended evidence was fused using the D-S combination rule to obtain the final fusion results as follows:

\begin{eqnarray*}\begin{array}{@{}l@{}} \displaystyle \Theta =\{ \{ A\} ,\{ B\} ,\{ C\} \} \\ \displaystyle m=\{ 0.9290\,0.0014\,0.0700\} . \end{array} \end{eqnarray*}

The following fusion method of this paper is compared with other evidence fusion methods in Table 1.

Observing the above five evidences, it can be seen that the probability that evidences E1, E3, E4 and E5 point to the focal element A is not less than 0.5. However, the probability that evidence E2 points to B is not less than 0.5, which conflicts with the other four evidences.

The analysis of the fusion results obtained by different methods in Table 1 shows that the D-S evidence theory cannot obtain correct fusion results when the evidence is in a highly conflicting or completely conflicting state. Both methods of Deng et al. (2005) and Ali, Dutta & Boruah (2012) can clearly identify the target only when the fourth piece of evidence is added. Additionally, the method of Deng et al. (2005) does not satisfy the exchange and combination laws of evidence fusion and has some limitations. Moreover, the study in Ali, Dutta & Boruah (2012) proposes a new evidence combination method, but it is not apparent for target identification results. The methods in both Tang et al. (2017) and Hu, Zhong & Liu (2016) use information entropy to deal with conflicting evidence and can identify the target, but the identification accuracy is not high when compared with the method proposed in this paper, and only one correction is made. Both Hu, Zhong & Liu (2016) and Tian, Ye & Wan (2021) use iterative methods to correct the evidence, which can accurately identify the target and have high recognition accuracy. However, the higher the accuracy requirement, the more iterations are needed, and the computation and workload are significantly increased; also, the computation is larger when dealing with larger-scale evidence. The comparative analysis shows that the proposed method can identify the target better. When the fourth piece of evidence is added *m*(*A*) = 0.8483, the results are more similar to the methods of Deng et al. (2005), Ye & Nie (2015), Tang et al. (2017) and Hu, Zhong & Liu (2016) when the fifth piece of evidence is added. When the fifth piece of evidence is added, the recognition rate of identifying target A is as high as 92.07%, which is higher than the recognition rate of the rest of the synthetic methods, and the accuracy of the method in this paper in identifying the target is higher when the evidence keeps increasing.

**Table 1:** Comparison of results of evidence fusion methods.

**Evidence fusion methodology**	**Objectives**	** ${\mathop{\oplus }\nolimits }_{i=1}^{3}{m}_{i}$ **	** ${\mathop{\oplus }\nolimits }_{i=1}^{4}{m}_{i}$ **	** ${\mathop{\oplus }\nolimits }_{i=1}^{5}{m}_{i}$ **
Petturiti & Vantaggi (2017)	A	0	0	0
	B	0.6316	0.3288	0.1228
	C	0.3684	0.6714	0.8772
Deng et al. (2005)	A	0.4861	0.7773	0.8909
	B	0.3481	0.0628	0.0086
	C	0.1657	0.1600	0.1005
Ali, Dutta & Boruah (2012)	A	0.3888	0.4797	0.5588
	B	0.3771	0.2375	0.1586
	C	0.2341	0.2828	0.2816
Ye & Nie (2015)	A	0.6900	0.8027	0.8759
	B	0.0610	0.0129	0.0030
	C	0.2490	0.1843	0.1211
Tang et al. (2017)	A	0.5079	0.7193	0.8937
	B	0.0135	0.1010	0.0003
	C	0.4786	0.1797	0.1060
Hu, Zhong & Liu (2016)	A	0	0.6954	0.7338
	B	0.8750	0.0230	0.0267
	C	0.1250	0.2816	0.2395
Tian, Ye & Wan (2021)	A	0.7176	0.8505	0.9150
	B	0.1311	0.0343	0.0022
	C	0.1513	0.1152	0.0828
Proposed methodology	A	0.6489	0.8483	0.9290
	B	0.1667	0.0141	0.0014
	C	0.1844	0.1376	0.0700

### Comparison of results of multi-method conflict evidence combination

The following will use several types of typical combination methods to synthesize the four types of typical conflict problems introduced in section 2.2 to compare with the methods in this paper, and the specific combination results are shown in Table 2.

**Table 2:** Comparison of results of evidence fusion methods.

**Type of paradox**			**Full conflict paradox (Example 1)**	**0 trust paradox** **(Example 2)**	**1 trust paradox** **(Example 3)**	**Evidence failure paradox (Example 4)**
**Combination method**						
1	Classic method	Petturiti & Vantaggi (2017)	Unable to synthesize	*m*(*B*) = 1	*m*(*B*) = 1	$\begin{array}{@{}l@{}} \displaystyle m(C)=0.35\\ \displaystyle m(C\cup D)=0.65 \end{array}$
2	Fix combination rules	Zhang, Pan & Zhang (2000)	$\begin{array}{@{}l@{}} \displaystyle L=0.5\\ \displaystyle m(A)=0.5\\ \displaystyle m(B)=0.5 \end{array}$	Unable to synthesize	Unable to synthesize	Unable to synthesize
3		Huang, Wei & Zhang (2018)	$\begin{array}{@{}l@{}} \displaystyle m(A)=0.5\\ \displaystyle m(B)=0.5 \end{array}$	$\begin{array}{@{}l@{}} \displaystyle m(A)=0.68\\ \displaystyle m(B)=0.02\\ \displaystyle m(C)=0.3 \end{array}$	$\begin{array}{@{}l@{}} \displaystyle m(A)=0.3333\\ \displaystyle m(B)=0.0001\\ \displaystyle m(C)=0.6666 \end{array}$	$\begin{array}{@{}l@{}} \displaystyle m(B)=0.65\\ \displaystyle m(C)=0.12\\ \displaystyle m(C\cup D)=0.23 \end{array}$
4	Amending the body of evidence	Han, Yang & Yuan (2010)	$\begin{array}{@{}l@{}} \displaystyle m(A)=0.5\\ \displaystyle m(B)=0.5 \end{array}$	$\begin{array}{@{}l@{}} \displaystyle m(A)=0.9957\\ \displaystyle m(C)=0.0043 \end{array}$	$\begin{array}{@{}l@{}} \displaystyle m(A)=0.33\\ \displaystyle m(B)=0.33\\ \displaystyle m(C)=0.01\\ \displaystyle m(D)=0.33 \end{array}$	$\begin{array}{@{}l@{}} \displaystyle m(B)=0.79\\ \displaystyle m(C)=0.141\\ \displaystyle m(C\cup D)=0.067\\ \displaystyle m(\Theta )=0.002 \end{array}$
5		Qian et al. (2021)	$\begin{array}{@{}l@{}} \displaystyle m(A)=0.5\\ \displaystyle m(B)=0.5 \end{array}$	$\begin{array}{@{}l@{}} \displaystyle m(A)=0.99\\ \displaystyle m(B)=0.002\\ \displaystyle m(C)=0.008 \end{array}$	$\begin{array}{@{}l@{}} \displaystyle m(A)=0.33\\ \displaystyle m(B)=0.33\\ \displaystyle m(C)=0.01\\ \displaystyle m(D)=0.33 \end{array}$	$\begin{array}{@{}l@{}} \displaystyle m(B)=0.86\\ \displaystyle m(C)=0.08\\ \displaystyle m(C\cup D)=0.04\\ \displaystyle m(\Theta )=0.02 \end{array}$
6	Both body of evidence and combination rules were amended	Cao et al. (2006)	$\begin{array}{@{}l@{}} \displaystyle \omega =0.5\\ \displaystyle m(A)=0.5\\ \displaystyle m(B)=0.5 \end{array}$	Unable to synthesize	Unable to synthesize	Unable to synthesize
7	Improved methodology based on open framework	Xu et al. (2007)	$m(\overline{\varphi })=1$	$\begin{array}{@{}l@{}} \displaystyle m(A)=0.774\\ \displaystyle m(B)=0.101\\ \displaystyle m(C)=0.155 \end{array}$	$m(\overline{\varphi })=1$	$\begin{array}{@{}l@{}} \displaystyle m(B)=0.8\\ \displaystyle m(C\cup D)=0.2 \end{array}$
8		Guo, He & Li (2015)	*m*(Θ) = 1	$\begin{array}{@{}l@{}} \displaystyle m(A)=0.504\\ \displaystyle m(B)=0.001\\ \displaystyle m(C)=0.018\\ \displaystyle m(A\cup B)=0.019\\ \displaystyle m(B\cup C)=0.003\\ \displaystyle m(A\cup C)=0.186\\ \displaystyle m(\Theta )=0.266 \end{array}$	$\begin{array}{@{}l@{}} \displaystyle m(B)=0.000001\\ \displaystyle m(A\cup B)=0.000099\\ \displaystyle m(B\cup C)=0.000099\\ \displaystyle m(A\cup B\cup C)=0.009801\\ \displaystyle m(B\cup D)=0.000099\\ \displaystyle m(A\cup B\cup D)=0.009801\\ \displaystyle m(A\cup C\cup D)=0.970299\\ \displaystyle m(B\cup C\cup D)=0.009801 \end{array}$	$\begin{array}{@{}l@{}} \displaystyle m(C)=0.014\\ \displaystyle m(B\cup C)=0.336\\ \displaystyle m(C\cup D)=0.026\\ \displaystyle m(B\cup C\cup D)=0.624 \end{array}$
9	Amending body of evidence	Combination method in this study	$\begin{array}{@{}l@{}} \displaystyle m(A)=0.5\\ \displaystyle m(B)=0.5 \end{array}$	$\begin{array}{@{}l@{}} \displaystyle m(A)=0.9878\\ \displaystyle m(B)=0.0005\\ \displaystyle m(C)=0.0117 \end{array}$	$\begin{array}{@{}l@{}} \displaystyle m(A)=0.33\\ \displaystyle m(B)=0.01\\ \displaystyle m(C)=0.33\\ \displaystyle m(D)=0.33 \end{array}$	$\begin{array}{@{}l@{}} \displaystyle m(B)=0.7839\\ \displaystyle m(C)=0.0001\\ \displaystyle m(C\cup D)=0.0006\\ \displaystyle m(\Theta )=0.2154 \end{array}$

After analyzing Table 2, according to the description in section 2.2, it can be seen that for example 1 the reasonable fusion result is 1 > *m*(*A*) = *m*(*B*) > 0, the traditional D-S combination rule cannot be fused, the open-framework-based improvement method is inconsistent with this criterion, and the above two methods cannot deal with the full conflict paradox. For example 2, the reasonable fusion result is 1 > *m*(*A*) > *m*(*C*) > *m*(*B*) > 0; according to this criterion, it can be seen that the traditional D-S combination rule, absorption method, weighted average method, and weighted distribution conflict method are obviously inconsistent with this criterion, and the above methods cannot deal with the 0 trust paradox. For example 3, the reasonable fusion result is 1 > *m*(*A*) = *m*(*C*) = *m*(*D*) > *m*(*B*) > 0. According to this criterion, only the method proposed in this paper, weighted average method, and conflicting evidence combination method based on the similarity coefficient between evidences can deal with the 1 trust paradox in Table 2; the rest of the methods cannot deal with the 1 trust paradox. For example 4, the reasonable fusion result is 1 > *m*(*B*) > *m*(*C*∪*D*) > *m*(*C*) > 0 and *m*(*B*) < *m*_2_(*B*) = *m*_1_(*B*), *m*(*C*∪*D*) < *m*_1_(*C*∪*D*), *m*(*C*) < *m*_1_(*C*). According to this criterion the traditional D-S combination rule, absorption method, weighted allocation conflict method, and open frame of discernment combination rule based on evidence distance in Table 2 cannot deal with the paradox of evidence failure. According to the combination results in Table 2, the reasonable situation of each combination method can be determined, and only the conflicting evidence combination method based on the similarity coefficient between evidences and the method in this paper can solve the main problems of D-S evidence theory. In the following section, the advantages and disadvantages of the proposed method and conflicting evidence combination method based on inter-evidence similarity coefficients will be further compared and analyzed through multiple conflicting evidence combinations.

### Comparative analysis of results of multi-conflict evidence combination

For the frame of discernment Θ = *A*, *B*, *C*, *D* a sensor collects seven mutually independent evidences whose corresponding basic probability assignments are as follows:

\begin{eqnarray*} \left\{ \begin{array}{@{}l@{}} \displaystyle {E}_{1}:{m}_{1}(A)=0.9,{m}_{1}(B)=0.1,{m}_{1}(C)=0,{m}_{1}(D)=0\\ \displaystyle {E}_{2}:{m}_{2}(A)=0.5,{m}_{2}(B)=0.5,{m}_{2}(C)=0,{m}_{2}(D)=0\\ \displaystyle {E}_{3}:{m}_{3}(A)=0.1,{m}_{3}(B)=0.9,{m}_{3}(C)=0,{m}_{3}(D)=0\\ \displaystyle {E}_{4}:{m}_{4}(A)=0,{m}_{4}(B)=0.6,{m}_{4}(C)=0.4,{m}_{4}(D)=0\\ \displaystyle {E}_{5}:{m}_{5}(A)=0,{m}_{5}(B)=0.9,{m}_{5}(C)=0,{m}_{5}(D)=0.1\\ \displaystyle {E}_{6}:{m}_{6}(A)=0,{m}_{6}(B)=0.5,{m}_{6}(C)=0.5,{m}_{6}(D)=0\\ \displaystyle {E}_{7}:{m}_{7}(A)=0,{m}_{7}(B)=0,{m}_{7}(C)=0.1,{m}_{7}(D)=0.9 \end{array} \right. \end{eqnarray*}

Data source from literature (Chen & Wang, 2021).

The combination results obtained using the proposed method in this paper and conflicting evidence combination method based on the similarity coefficients between evidences were synthesized separately as shown in Table 3.

For the focal element X, the size of its synthetic basic probability assignment *m*(*X*) isinfluenced by two aspects. The first is the overall support of the individual evidence for the focal element, which can be quantified by the concept of basic confidence number, *i.e.,* $\bar {{m}_{i}(X)}= \frac{\sum {m}_{i}(X)}{N} $, where *N* is the number of evidences. The second is the number of pieces of evidence supporting the focal element X.

Observing the above seven pieces of evidence, we can see that *E*_2_, *E*_3_, *E*_4_, *E*_5_, *E*_6_ all point to element B with a high probability. The basic probability assignment of evidence *E*_2_, *E*_3_ to focal element A is the same as that of evidence *E*_6_, *E*_7_ to focal element C. However, the synthesis result of *m*_1_(*A*) > *m*_4_(*C*)is *m*(*A*) > *m*(*C*) according to common sense. The evidences *E*_4_, *E*_6_, *E*_7_ support 0.4, 0.5, and 0.1 for focal element C, respectively, and evidences *E*_5_, *E*_7_ support 0.1, 0.9, and 0.9 for focal element D, respectively. The average basic credible number of both of them is the same, but the number of evidence supporting focal element C is greater than the number of evidence supporting focal element D. Then, according to common sense, its synthetic result is *m*(*C*) > *m*(*D*). In summary, its synthetic result is the reasonable standard *m*(*B*) > *m*(*A*) > *m*(*C*) > *m*(*D*). The analysis of Table 3 shows that the combination results of the conflicting evidence combination method based on the similarity coefficient between evidences do not meet the reasonable criteria. The method proposed in this paper not only meets the reasonable criteria, but can also identify target B with a higher probability. Therefore, the proposed method is more reasonable.

**Table 3:** Comparison of results of evidence fusion methods.

**Combination method**	**Proposed method**	**Conflicting evidence combination method based on similarity coefficient between evidences** (Qian et al., 2021)
*m*(*A*)	0.00054	0.0003
*m*(*B*)	0.99875	0.9996
*m*(*C*)	0.00049	0.00005
*m*(*D*)	0.00022	0.00005

### Comparative analysis of evidence synthesis results in practical applications

To show the effectiveness of the method proposed in this paper in practical applications, an example of missile health status assessment in literature (Li, Liu & Bao, 2022) was used for comparative analysis. Taking a certain type of surface-to-air missile as an example, assume that the test index characterizing its health status is *p*_1_, *p*_2_, *p*_3_, *p*_4_, *p*_5_. In one of the tests in 2020, if it appears that the actual measured value of a test parameter is outside the standard threshold range, the health status of the device is directly determined to be a fault state. Taking one of the missiles as an example, the actual value of the current test, the non-faulty value of the last test, the average value of the historical test, and the standard threshold value for the five test parameters are shown in Table 4.

Assuming that the quality levels corresponding to excellent, very good, good and proposed failure are A, B, C and D, respectively, the BPA is constructed according to the method proposed in the literature (Li, Liu & Bao, 2022). The test parameter *p*_1_, *p*_2_, *p*_3_, *p*_4_, *p*_5_ corresponds to a BPA of *m*_1_, *m*_2_, *m*_3_, *m*_4_, *m*_5_, and the specific values are shown in Table 5.

The method proposed in this paper is combined with other conflict evidence synthesis methods respectively, and the results are shown in Table 6.

Since the value of *p*_1_ for this test is 0. 69, *i.e.,* outside the threshold range, there is a certain degree of conflict in the test message. The data in Table 6 are all four iterations of fusion results, from the fusion results in Table 6 we can see that the missile health state is in a very good state, due to the existence of conflicting evidence, the D-S algorithm can not handle conflicting evidence, so the fusion results can not be obtained, the actual measured value of parameter 1 is about to exceed the standard threshold range, although the overall health state of the missile tends to be good, but if there is an abnormality in a certain test index, in the synthesis, the information should be retained as much as possible to ensure that the synthesis results are true and valid, Then the synthesis results of literature (Kleinberg, 1999)and literature (Gao, Pan & Deng, 2021) have some deviation from the actual situation. The proposed method in this paper has high accuracy of target identification compared to literature (Murphy, 2000), literature (Li, Liu & Bao, 2022), and also retains the information of the existence of anomalies in the test index when fusion.

**Table 4:** Single missile test parameters.

Test parameters	Value of this test	Last test value	Historical average	Standard value	Error limits	Upper thresholds	Lower thresholds
*p*_1_	0.6900	0.540	0.450	0.50	±0.20	0.7	0.3
*p*_2_	26.800	28.089	27.120	27.00	±2.00	29.5	24.5
*p*_3_	48.139	46.834	46.945	47.15	±1.85	49.0	45.3
*p*_4_	10.528	11.583	10.346	11.00	±1.00	12.0	10.0
*p*_5_	78.640	80.200	79.754	80.00	±2.00	82.0	78.0

**Table 5:** Basic probability assignment.

Quality level	*m*_1_	*m*_2_	*m*_3_	*m*_4_	*m*_5_
*A*	0	0	0	0.0194	0.2765
*B*	0	0.9881	0.9993	0.9806	0.7235
*C*	0.8965	0.0119	0.0007	0	0
*D*	0.1035	0	0	0	0

**Table 6:** Comparison of fusion results.

Name of algorithm	*m*(*A*)	*m*(*B*)	*m*(*C*)	*m*(*D*)
Petturiti & Vantaggi (2017)	Unable to synthesize	Unable to synthesize	Unable to synthesize	Unable to synthesize
Murphy (2000)	0	0.99909	0.00091	0
Kleinberg (1999)	0	1	0	0
Li, Liu & Bao (2022)	0	0.99980	0.00020	0
Gao, Pan & Deng (2021)	0	1	0	0
Proposed methodology	0	0.99999	0.00001	0

## Conclusions

The traditional D-S combination rule cannot handle highly conflicting evidence and produces contradiction with facts in practical applications. To address this shortcoming, this paper proposes an evidence combination method based on SALSA algorithm and Lance-Williams distance. The verification simulation shows that the proposed method can effectively deal with highly conflicting evidence compared with the traditional D-S combination rule, with faster recognition speed, while reducing the uncertainty of recognition results. The shortcomings of the proposed method in this paper is that it only considers the relationship between the evidence and does not take into the influence of internal evidence on evidence preprocessing methods, The next step in the research plan is to consider addressing this shortcoming.

##  Supplemental Information

10.7717/peerj-cs.1307/supp-1 Supplemental Information 1 Coefficient of association

10.7717/peerj-cs.1307/supp-2 Supplemental Information 2 Evidence of correction

10.7717/peerj-cs.1307/supp-3 Supplemental Information 3 C file used to calculate the Lance&Williams distance between various evidences

## Additional Information and Declarations

### Data Availability

The following information was supplied regarding data availability: The code is available in the Supplemental Files.

## References

[ref-1] Ali T, Dutta P, Boruah H (2012). A new combination rule for conflict problem of dempster-shafer evidence theory. International Journal of Energy, Information and Communications.

[ref-2] Bo XY, Chen XY, Li HS, Dong YC, Qu ZY, Wang L, Li Y (2021). Modeling method for the coupling relations of microgrid cyber-physical systems driven by hybrid spatiotemporal events. IEEE Access.

[ref-3] Brin S, Page L (1998). The anatomy of a large-scale hypertextual web search engine. Computer Networks and ISDN Systems.

[ref-4] Cao LJ, Qin JQ, Ma JS, Wang XG (2006). Research on parallel reasoning mechanism based on multi-agent in fault diagnosis system. Computer Measurement and Control.

[ref-5] Chen Z, Wang JY (2021). Research on evidence synthesis method based on conflict relationship network. Journal of Electronics.

[ref-6] Deng Y, Shi WK, Zhu ZF, Liu Q (2005). Combining belief functions based on distance of evidence. Decision Support Systems.

[ref-7] Ding H, Hou RC, Ding XQ (2019). A fault diagnosis method based on rough set and improved D-S evidence theory. Computer and Digital Engineering.

[ref-8] Gao XZ, Pan LP, Deng Y (2021). Quantum Pythagorean Fuzzy Evidence Theory (QPFET): a negation of quantum mass function view. IEEE Transactions on Fuzzy Systems.

[ref-9] Guo Q, He Y, Li XD (2015). A fast DSmT-DS approximate inference fusion method. Journal of Electronics and Information.

[ref-10] Han F, Yang WH, Yuan XG (2010). A combinatorial approach for effective handling of conflicting evidence. Electro-Optics and Control.

[ref-11] Hu CH, Si SS, Zhou ZJ, Wang P (2009). An improved D-S algorithm with a new evidence conflict measure. Journal of Electronics.

[ref-12] Hu HL, Zhong QX, Liu L (2016). An improved approach to D-S evidence theory based on iterative synthesis. Computer Application Research.

[ref-13] Huang XZ, Wei DJ, Zhang CX (2018). An improved approach to the synthesis of conflicting evidence. Journal of Hubei Academy of Nationalities: Natural Science Edition.

[ref-14] Kleinberg JM (1999). Authoritative sources in a hyperlinked environment. Journal of the ACM.

[ref-15] Lempel R, Moran S (2000). The stochastic approach for link-structure analysis (SALSA) and the TKC effect. Computer Networks.

[ref-16] Li BC, Wang B, Wei J, Qian ZB, Huang YQ (2002). An effective evidence-theoretic synthesis formula. Data Acquisition and Processing.

[ref-17] Li XQ, Liu Y, Bao XY (2022). Application of D-S evidence theory in missile health status assessment. Measurement and Control Technology.

[ref-18] Murphy CK (2000). Combining belief functions when evidence conflicts. Decision Support Systems.

[ref-19] Petturiti D, Vantaggi B (2017). Upper and lower conditional probabilities induced by a multivalued mapping. Journal of Mathematical Analysis and Applications.

[ref-20] Qian JB, Wang XK, Dong J, Han W, Feng ZC (2021). A D-S fusion algorithm based on improved evidence similarity. Radio Engineering.

[ref-21] Shafer G (2020). A mathematical theory of evidence.

[ref-22] Sun Q, Ye XQ, Gu WK (2000). A new synthetic formulation based on evidence theory. Journal of Electronics.

[ref-23] Tang YY, Zhou DD, Xu S, He ZZ (2017). A weighted belief entropy-based uncertainty measure for multi-sensor data fusion. Sensors.

[ref-24] Tian MM, Ye JH, Wan YJ (2021). An iterative modified conflict evidence improvement method. Control Engineering.

[ref-25] Xu PL, Yang FB, Wang XX, Tan JY (2007). Research on D-S synthesis rules for open recognition framework. Sensors and Microsystems.

[ref-26] Xu YX, Li ZQ, Gu JY, Cong LH, An J, Zhao JZ (2018). Missile quality state assessment based on multi-state Bayesian networks. Journal of Military Engineering.

[ref-27] Yager RR (1987). On the Dempster-Shafer framework and new combination rules. Information Sciences.

[ref-28] Ye JH, Nie XS (2015). Application of improved evidence classification synthesis method in crop growth environment assessment. Data Collection and Processing.

[ref-29] Yong D, Li Q, Zhang YJ (2011). A new approach to conflict of evidence analysis. Control Theory and Applications.

[ref-30] Zhang GH, Duan MY, Zhang JH, Chen DG, Yang JY (2009). Risk assessment of power system based on evidence theory and utility theory. Power System Automation.

[ref-31] Zhang SY, Pan Q, Zhang HC (2000). A new combination rule for evidential reasoning. Control and Decision Making.

